# Does transcranial direct current stimulation improve functional locomotion in people with Parkinson’s disease? A systematic review and meta-analysis

**DOI:** 10.1186/s12984-019-0562-4

**Published:** 2019-07-08

**Authors:** Hyo Keun Lee, Se Ji Ahn, Yang Mi Shin, Nyeonju Kang, James H. Cauraugh

**Affiliations:** 1 Division of Sport Science, Neuromechanical Rehabilitation Research Laboratory, Incheon National University, 119 Academy-ro, Yeonsu-gu, Incheon, South Korea; 2 Vector Biomechanics Inc., Yongin, South Korea; 3 Sport Science Institute, Incheon National University, Incheon, South Korea; 4 Department of Applied Physiology and Kinesiology, University of Florida, Gainesville, Florida USA

**Keywords:** Parkinson’s disease, Functional locomotion, Transcranial direct current stimulation, Meta-analysis

## Abstract

**Purpose:**

The purpose of this meta-analysis was to investigate the treatment effects of transcranial direct current stimulation (tDCS) on functional locomotion in people with Parkinson’s disease (PD).

**Methods:**

A systematic literature search identified 18 qualified studies that used tDCS protocols as functional locomotion rehabilitation interventions for people with PD. All included studies used either a randomized control trial or crossover designs with a sham control group. Meta-analysis quantified both (a) short-term treatment effects: change in functional locomotion between baseline and immediate posttests on 18 comparisons and (b) long-term treatment effects: change in functional locomotion between baseline and delayed retention tests on six comparisons. Moreover, we performed moderator variable analyses for comparing effect sizes between tDCS targeting multiple brain regions and tDCS targeting a single brain region.

**Results:**

Random effects model meta-analyses revealed a significant short-term treatment effect (effect size = 0.359; *P* = 0.001), whereas no significant long-term treatment effects were identified (effect size = 0.164; *P* = 0.314). In addition, tDCS protocols that targeted multiple brain regions showed relatively more positive effects on functional locomotion than protocols that targeted a single brain region.

**Conclusions:**

These meta-analytic findings indicate that tDCS protocols may show immediate positive effects on functional locomotion in people with PD. However, given the relatively low effect size, exploring more appropriate tDCS protocols (i.e., targeting multiple motor and prefrontal regions and medication condition) should be a focus in future studies.

**Electronic supplementary material:**

The online version of this article (10.1186/s12984-019-0562-4) contains supplementary material, which is available to authorized users.

## Background

Parkinson’s disease (PD) is a neurodegenerative disease attributed to progressive degeneration of dopamine-producing neurons within the basal ganglia mainly affecting the motor cortex [1]. The cardinal symptoms of PD are manifested as motor related features including bradykinesia, rigidity, resting tremor, postural instability, and gait disturbance [2]. Applying medications such as levodopa and carbidopa, chemical supplements for increasing dopamine, may be an efficient clinical option for improving rigidity and slowness of movement of people with PD [3]. However, the pharmacological treatments are less effective as the disease progresses [4, 5]. Deep brain stimulation (DBS), a surgical approach, has been introduced and complementally used particularly for people with PD with moderate to severe disease severity [6]. Despite the evidence of treatment effects on motor symptoms of PD after DBS [7, 8], this neurosurgical option is cautiously used for PD treatment because of high cost and potential surgical risk [9, 10]. Therefore, exploring therapeutic alternatives and rehabilitation interventions as a complementary treatment is still required.

Recently, neurorehabilitation researchers have increased their attention on the utility of non-invasive brain stimulations as therapeutic alternatives for treating motor symptoms of PD [11]. In particular, transcranial direct current stimulation (tDCS) which is one of the non-invasive brain stimulation (NIBS) techniques has been investigated for PD motor recovery [12]. Despite insufficient findings regarding neurophysiological mechanisms underlying tDCS, this intervention may be an attractive rehabilitation option because of its practical advantages of economic efficiency, portability, and accessibility. Basically, tDCS provides both anodal and cathodal stimulations by delivering weak direct currents (e.g., intensity = 1-2 mA) to the scalp via surface electrodes. Based on potential mechanisms of tDCS that anodal tDCS increases cortical excitability and cathodal tDCS decreases cortical excitability [13], tDCS may reorganize neural activation patterns and facilitate neural plasticity in specific targeted regions of the brain [14, 15]. Perhaps, tDCS can potentially modulate functional connectivity among the cortico-striatal and thalamo-cortical circuits of brain [16]. These neuronal alterations by tDCS can provide functional advantages for PD motor rehabilitation. Moreover, Quartarone et al. [17] reported that the modulation of neuronal excitability may last beyond the stimulating period supporting the suggestion that tDCS may be effective for improving motor symptoms in people with PD.

Several studies using animal models showed tDCS findings modulating dopaminergic pathways [18, 19]. Specifically, anodal tDCS could activate dopaminergic neurons and promote dopamine levels of striatum in a monkey and rats with PD [18, 20]. These findings support the potential efficacy of tDCS in motor rehabilitation of people with PD [21]. For a human model, tDCS protocols primarily targeted motor and prefrontal cortices (e.g., primary motor cortex: M1 and dorsolateral prefrontal cortex: DLPFC) because brain activation patterns in these brain regions are highly involved in successful locomotion performance in people with PD [22–25]. Fregni et al. [22] suggested that greater M1 activation after anodal tDCS was related to improvements in motor function of PD. Further, people with PD revealed higher DLPFC activation during normal walking because they presumably compensated for deficits in gait automaticity by increasing cognitive control (e.g. executive control). Thus, more DLPFC activation by anodal tDCS may be necessary when people with PD completed more complex locomotion tasks [23–25]. Moreover, some repetitive transcranial magnetic stimulation (rTMS) studies reported release of dopamine in the caudate and putamen in healthy individuals [26, 27] and people with PD [28] when the stimulation triggered motor and prefrontal cortical regions. Presumably, applying tDCS may cause similar dopamine release contributing to acute motor improvements as well.

Two previous meta-analysis studies reported the overall positive effects of various NIBS techniques including rTMS, tDCS, and transcranial alternating current stimulation (tACS) on various motor symptoms in [29, 30]. However, the prior meta-analytic findings regarding motor improvement evidence of people with PD were estimated by heterogeneous outcome measures (e.g., tremor, rigidity, gait, and bradykinesia) and different NIBS protocols. Importantly, a recent systematic review study by Broeder et al. [31] suggested potential treatment effects of tDCS protocols on gait performance in people with PD. Locomotion impairment is one recognizable motor symptom compromising independence and quality of life in people with PD, and effectively represents an individual’s progression of disease severity [8]. Although a recent meta-analysis by Goodwill et al. [29] reported significant positive effects of tDCS and tACS on gait functions, these meta-analytic findings were still limited to small sample sizes (i.e., two studies). Thus, the current systematic review and meta-analysis investigated the treatment effects of tDCS on functional locomotion in people with PD. Moreover, given that multiple cerebral regions related to motor and cognitive functions may influence functional locomotion, we addressed an additional question: Do tDCS protocols targeting multiple brain regions and a single brain region reveal similar treatment effects on functional locomotion?

## Methods

### Literature search and study selection

The current meta-analyses were conducted consistent with the suggestions by the Preferred Reporting Items for Systematic Reviews and Meta-Analyses (PRISMA) statement consisting of a checklist and a flow diagram [32]. Especially, this study reported all PRISMA checklist items (Additional file 1), and failed to register the systematic review protocol because we already completed data extraction and analyses. We conducted a computerized literature search within July 2018–May 2019 using PubMed, Web of Science, and Cochrane Databased of Systematic Reviews. All types of publications were considered regardless of publication date. Search terms were: (a) Parkinson or Parkinson’s disease or PD, (b) transcranial direct current stimulation or transcranial electrical stimulation or tDCS, and (c) gait or walk or walking or locomotion or locomotor task. The inclusion criteria of this meta-analysis included: (a) reporting quantitative data related to functional locomotion, (b) including between-group comparisons (i.e., active tDCS versus sham tDCS), and (c) using either a crossover design or randomized control trial design.

### Extraction of functional locomotion outcome measures

We analyzed functional locomotion by investigating individual’s temporal components (i.e., gait speed and time to complete specific task) in various functional locomotion tests. Moreover, we estimated the treatment effects of tDCS on functional locomotion in PD for two different perspectives: (a) short-term effects indicating changes in functional locomotion (i.e., retention time between baseline and immediate posttest ≤24 h after final tDCS intervention) and (b) long-term effects denoting changes in functional locomotion (i.e., retention time between baseline and follow-up tests ≥4 weeks after final tDCS intervention).

### Methodological quality assessments

Two authors (SA and YS) independently assessed the methodological quality for the qualified studies using the Physiotherapy Evidence Database (PEDro) rating scale [33], and further estimated the risk of bias of all studies using the Cochrane risk of bias assessment [34]. The PEDro scale estimated study quality using a checklist of 10 items scored yes-or-no related to group allocation, blinding, attrition, statistical analyses, and data variability. Using Review Manager 5.3 software (Copenhagen: The Nordic Cochrane Centre, The Cochrane Collaboration, 2014), we additionally performed the Cochrane risk of bias assessment estimating (a) random sequence allocation, (b) allocation concealment, (c) blinding of participants and personnel, (d) blinding of outcome assessment, (e) incomplete outcome data, (f) selective reporting, and (g) other sources of bias. Discrepancies in quality assessment scores between the two authors activated a third assessor (NK) who separately graded and confirmed the final scores.

### Meta-analytic techniques

We conducted the meta-analyses using the Comprehensive Meta-Analysis software (ver. 3.0. Englewood, NJ, USA). For quantifying individual effect sizes, we calculated standardized mean difference (*SMD*) and 95% confidence intervals (CIs) for each comparison. SMDs and CIs for 18 short-term treatment comparisons and six long-term treatment comparisons were estimated. The current meta-analyses used a random effects model because we posited that individual effect sizes are different and no common effect size across comparisons exists because of different participant populations, treatment protocols, and statistical designs [35]. In addition, we conducted a moderator variable analysis quantifying overall effect sizes between two sub-groups: (a) tDCS targeting multiple brain regions versus (b) tDCS targeting a single brain region. However, we examined the moderator variable analysis for the short-term treatment effects of tDCS only because of insufficient number of comparisons for the long-term treatment effects of tDCS.

To quantify variability of individual effect sizes across qualified studies, we performed three heterogeneity tests: (a) Cochran’s *Q*, (b) *T*^2^ (estimate of tau-squared) and (C) Higgins and Green’s *I*^2^. Cochran’s *Q* test provides Q statistics with *P*-value at alpha level equal to 0.05. *P*-value less than 0.05 indicated a significant heterogeneity across studies [35]. The level of *T*^2^ is an estimate of variance of the observed effects with weights assigned in a random effects model so that *T*^2^ greater than 1.0 denotes a significant level of variability across studies [36, 37]. Finally, *I*^2^ is the percentage of the heterogeneity, and further *I*^2^ higher than 50% indicates substantial between-studies heterogeneity [38].

The publication bias indicating the risk of bias across studies was estimated with three techniques: (a) funnel plot, (b) Egger’s regression test, and (c) Begg and Mazumdar rank correlation test. One conventional approach is to visually estimate publication bias level via the funnel plot displaying *SMD* vs. standard error for each comparison. Before and after applying trim and fill technique [39], we compared two overall effect sizes between the original plot and the revised plot with potential imputed values. Lower changes in the overall effect sizes with minimum number of imputed values potentially indicates a minimal level of publication bias across studies. Egger’s regression and Begg and Mazumdar rank correlation tests are quantitative approaches to measuring the level of publication bias. Egger’s regression test provides the asymmetry of funnel plot by calculating the intercept from regression of standard normal deviates versus precision so that an intercept with *P*-value greater than 0.05 indicates minimal publication bias [40]. Similarly, Begg and Mazumdar rank correlation test shows the correlation between the ranks of effect sizes and the ranks of their variances. Thus, the rank correlation (Kendall’s Tau) with *P*-values greater than 0.05 denotes minimal risk of bias across studies [41].

## Results

### Study identification

**Fig. 1:**
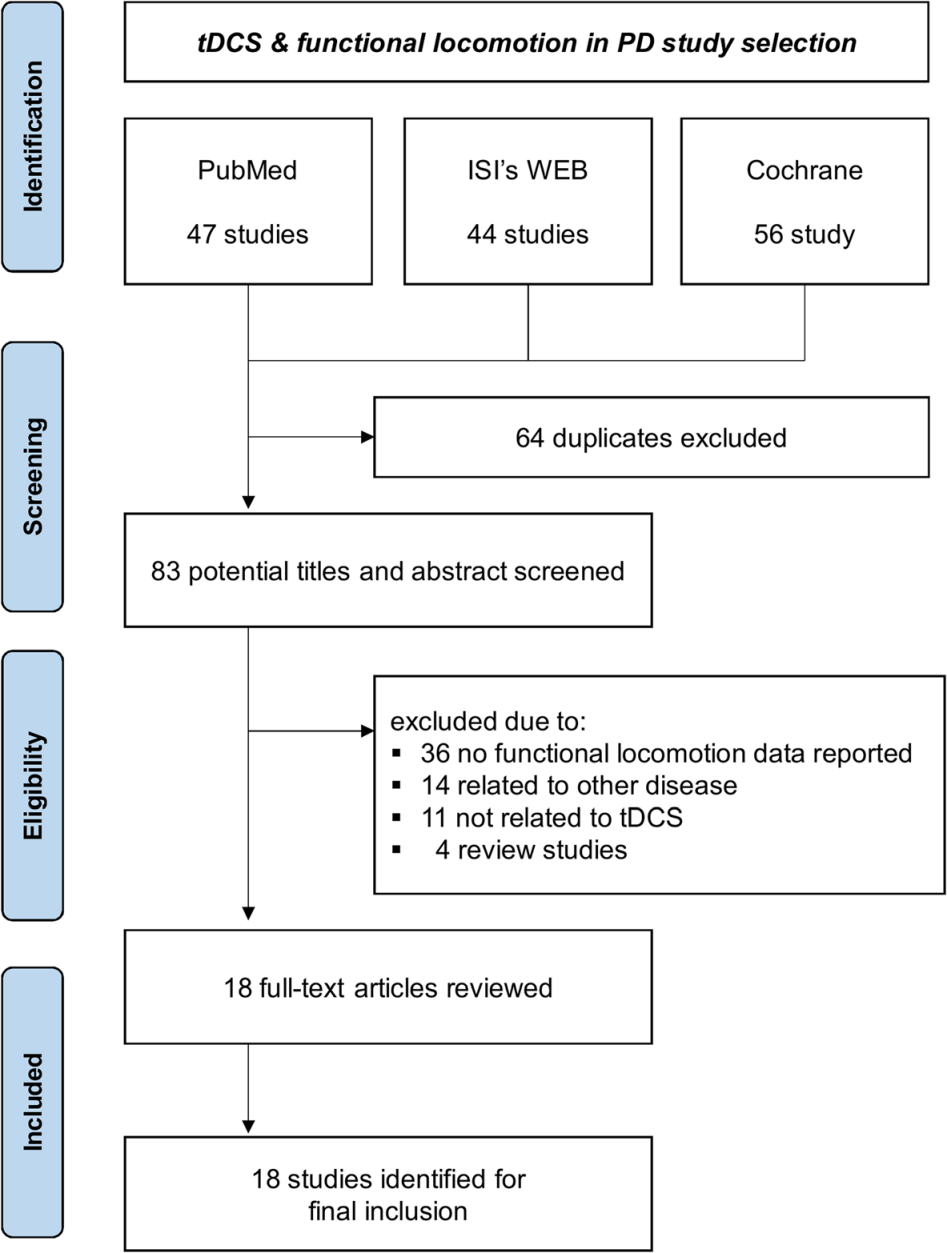
PRISMA flow diagram

The PRISMA flow diagram in Fig. 1 shows the specific steps for the study identification procedures. Initially, our literature search identified 147 potential studies, and excluded 64 duplicated articles. After reviewing the abstract and text for each study based on our inclusion and exclusion criteria by three authors (NK, SA, and YS), we excluded 65 studies because of review articles, not-related disease, no functional locomotion results reported (e.g., protocol study), and not applying tDCS. Finally, 18 total studies met our inclusion criteria were qualified for the meta-analysis [42–59].

### Participant characteristics

The 18 qualified studies included 325 people with PD (range of mean age = 56.7–72.3 years). A range of mean period beyond PD diagnosis was 4.6–16.8 years. A range of mean motor examination scores in The Unified Parkinson’s Disease Rating Scale at baseline equaled 11.2–47.7 (higher scores indicate worse motor functions). Fourteen out of 18 total studies reported medication status of participants that all people with PD were on medication, and the remaining four studies did not mention medication status. Specific details regarding participant characteristics are shown in Table 1.

**Table 1:** Participant characteristics

Study	Total N	Age (yrs)	Gender	PD Duration (yrs)	UPDRS Part III at Baseline	Medication	DBS Treatment	FOG Test
Alizad [42]	8	NA	Total: 3F, 5 M	NA	NA	NA	NO	NA
Benninger [43]	25	Total: 63.9 ± 8.7	Active: 4F, 9 M Sham: 5F, 7 M	Active: 10.6 ± 7.1 Sham: 9.1 ± 3.3	Active: 22.2 ± 8.7 Sham: 17.5 ± 8.0	On	NO	Patients with severe freezing or unable to walk 10 m were excluded
Capacci [44]	7	Total: 60.9 ± 9	Total: 4F, 3 M	Total: 16.8 ± 4.0	NA	NA	NO	NA
Costa-Ribeiro [45]	22	Active: 61.1 ± 9.1; Sham: 62.0 ± 16.7	Active: 3F, 8 M Sham: 4F, 7 M	Active: 6.1 ± 3.8 Sham: 6.3 ± 3.7	Active: 19.0 Sham: 19.1	On	NO	FOG-Q(> 15 points) were excluded
Costa-Ribeiro [46]	22	Active: 61.1 ± 9.1 Sham: 62.0 ± 16.7	Active: 3F, 8 M Sham: 4F, 7 M	Active: 6.1 ± 3.8 Sham: 6.3 ± 3.7	Active: 19.0 ± 4.9 Sham: 17.6 ± 5.1	On	NO	Patients were excluded when they presented severe freezing according the FOG-Q
Criminger [47]	16	Total: 68.1 ± 9.8	Total: 4F, 12 M	Total: 8.7 ± 9.8	Total: 23.4 ± 9.7	On	NO	NA
da Silva [48]	17	Active: 66.0 ± 5.0 Sham: 66.0 ± 10.0	Active: 4F, 4 M Sham: 3F, 6 M	Active: 6.0 ± 6.0 Sham: 5.0 ± 1.0	NA	NA	NO	NA
Dagan [49]	20	NA	NA	NA	NA	On	NO	FOG-Q: 20.5 ± 4.9 FOG-provoking test scores: 14.2 ± 8.00
Fernández-Lago [50]	18	Total: 56.7 ± 11.6	Total: 7F, 11 M	Total: 6.2 ± 3.7	Total: 21.17 ± 11.3	On	NO	NA
Kaski [51]	16	NA	NA	NA	NA	On	NO	Patients with severe freezing were excluded
Lattari [52]	17	Total: 67.2 ± 10.0	Total: 4F, 13 M	Total: 7.1 ± 2.7	Total: 18.0 ± 99.0	On	NO	NA
Mak [53]	18	NA	NA	NA	NA	NA	NO	NA
Manenti [54]	10	Total: 67.1 ± 7.2	Total: 4F, 6 M	Total: 8.1 ± 3.5	Total: 13.3 ± 5.7	On	NO	NA
Schabrun [55]	16	Active: 72.0 ± 4.9 Sham: 63.0 ± 11.0	Active: 8 M Sham: 6F, 2 M	Active: 6.9 ± 4.4 Sham: 4.6 ± 3.9	Active: 47.7 ± 7.5 Sham: 37.7 ± 9.8	On	NO	NA
Swank [56]	10	Total: 68.7 ± 10.2	Total: 2F, 8 M	Total: 7.9 ± 7.1	Total: 37.0 ± 12.9	On	NO	NA
Valentino [57]	10	Total: 72.3 ± 3.6	Total: 5F, 5 M	Total: 11.0 ± 4.9	Total: 32.0 ± 10.3	On	NO	FOG-Q: 15.3 ± 2.7
Verheyden [58]	20	NA	NA	Total: 9.0 ± 4.0	Total: 16.0 ± 5.0	On	NO	NA
Yotnuengnit [59]	53	Active: 68.2 ± 9.8 Sham: 62.7 ± 8.8	Active: 6F, 11 M Sham: 6F, 12 M	Active: 9.4 ± 5.3 Sham: 6.6 ± 3.6	Active: 11.9 ± 4.7 Sham: 11.2 ± 4.0	On	NO	NA

*Abbreviations*: *Active* Active tDCS protocols, *DBS* Deep brain stimulation, *F* Female, *FOG* Freezing of gait, *FOG-Q* Freezing of gait questionnaire, *M* Male, *NA* Not applicable, *PD Duration* Time since PD diagnosis, *UPDRS* The Unified Parkinson’s Disease Rating Scale. Note. Data for age and PD duration are mean ± standard deviation

### tDCS intervention protocols

Table2 shows specific tDCS parameters for the qualified studies. All 18 included studies used active tDCS (i.e., anodal tDCS: 16 studies and anodal & cathodal tDCS: two studies) and sham stimulation. Targeted brain regions of active tDCS for the qualified studies included prefrontal cortex (PFC), dorsal lateral prefrontal cortex (DLPFC), premotor cortex (PMC), supplementary motor area (SMA), primary motor cortex (M1; C3 or C4 in the International 10–20 system), and leg areas of M1 (Cz in the International 10–20 system). Six studies stimulated multiple brain regions (e.g., one bilateral PFC and M1; one bilateral PFC, PMC, and M1; one bilateral PMC and M1; two bilateral DLPFC; one M1 and left-DLPFC) whereas 12 studies used a single targeted brain region. Six out of 12 studies that used a single targeted brain region applied anodal tDCS on the central leg areas of M1, and the remaining six studies targeted either M1 or DLPFC of one side of hemisphere (one leg area of M1 of affected hemisphere; three M1 of left hemisphere; one left DLPFC; one right DLPFC). Finally, seven studies used a single session of tDCS protocols and 11 studies applied multiple sessions of tDCS protocols.

**Table 2:** tDCS protocols

Study	Treatment	Session #	Active tDCS	Stimulation Site	Stimulation Parameters (Intensity, Duration, Areas)	Follow-Up Test
Alizad [42]	tDCS	3	A	M: Bi PMC & M1	1 mA, 20 min, 40 cm^2^	No
Benninger [43]	tDCS	8	A	M: Bi PFC, PMC, & M1 (separately)	2 mA, 20 min, 24.5 cm^2^	Yes (12wks)
Capacci [44]	tDCS	1	A	M: Bi PFC (separately)	2 mA, 20 min, NA	No
Costa-Ribeiro [45]	tDCS&GT	10	A	S: Central leg areas M1 (2 cm anterior to the vertex)	2 mA, 13 min, NA	Yes (4wks)
Costa-Ribeiro [46]	tDCS&GT	10	A	S: Central leg areas M1 (2 cm anterior to the vertex)	2 mA, 13 min, 35 cm^2^	Yes (4wks)
Criminger [47]	tDCS	3	A&C	M: Bi DLPFC (A-tDCS on LH & C-tDCS on RH)	2 mA, 20 min, 15 cm^2^	No
da Silva [48]	tDCS	1	A	S: Central leg areas M1 & SMA	2 mA, 15 min, 35 cm^2^	No
Dagan [49]	tDCS	2	A	M: M1 & LH-DLPFC	20 min, NA, NA	No
Fernández-Lago [50]	tDCS&TT	1	A	S: leg area M1 of AH	2 mA, 20 min, 3.5 cm^2^	No
Kaski [51]	tDCS&PT	1	A	S: Central leg areas M1 (10–20% anterior to the vertex)	2 mA, 15 min, 40 cm^2^	No
Lattari [52]	tDCS	1	A	S: LH DLPFC	2 mA, 20 min, 35 cm^2^	No
Mak [53]	tDCS	5	A	S: M1	NA, 20 min, NA	No
Manenti [54]	tDCS	2	A	S: RH DLPFC	2 mA, 7 min, 35 cm^2^	No
Schabrun [55]	tDCS&GT	9	A	S: LH M1	2 mA, 20 min, 35 cm^2^	Yes (12wks)
Swank [56]	tDCS	1	A&C	M: Bi DLPFC (A-tDCS on LH & C-tDCS on RH)	2 mA, 20 min, NA	No
Valentino [57]	tDCS	5	A	S: Central leg areas M1	2 mA, 20 min, NA	Yes (4wks)
Verheyden [58]	tDCS	1	A	S: LH M1	1 mA, 15 min, NA	No
Yotnuengnit [59]	tDCS&PT	6	A	S: Central leg areas M1	2 mA, 30 min, 35 cm^2^	Yes (8wks)

*Abbreviations*: *A* Anodal tDCS, *AH* Affected hemisphere, *Bi* Bilateral, *C* Cathodal tDCS, *DLPFC* Dorsolateral prefrontal cortex, *GT* Gait training, *LH* Left hemisphere, *M* Multiple targeted brain regions, *M1* Primary motor cortex, *NA* Not applicable, *PFC* Prefrontal cortex, *PMC* Premotor cortex, *PT* Physical training, *RH* Right hemisphere, *S* Single targeted brain region, *TT* Treadmill training, *wks* Weeks (retention period)

### Functional locomotion outcome measures

Eighteen qualified studies reported one of following outcome measures: (a) gait speed: eight studies and (b) the time that a person takes to complete certain locomotion tasks: eight studies for Timed Up and Go test (TUG), one study for 10 m walking time, and one study for Stand Walk Sit test. All included studies reported short-term effects of tDCS on functional locomotion, and six studies of 18 total studies reported long-term effects (mean ± SD of retention time = 7.3 ± 3.9 weeks). For short-term effects of tDCS, seven studies reported functional locomotion difference between active and sham tDCS groups at posttest whereas 11 studies reported changes in functional locomotion between baseline and posttest after active tDCS as compared with sham stimulation. For long-term effects of tDCS, four studies reported functional locomotion difference between active and sham tDCS groups at posttest whereas two studies reported changes in functional locomotion between baseline and posttest after active tDCS as compared with sham stimulation.

### Methodological quality assessments over included studies

**Fig. 2:**
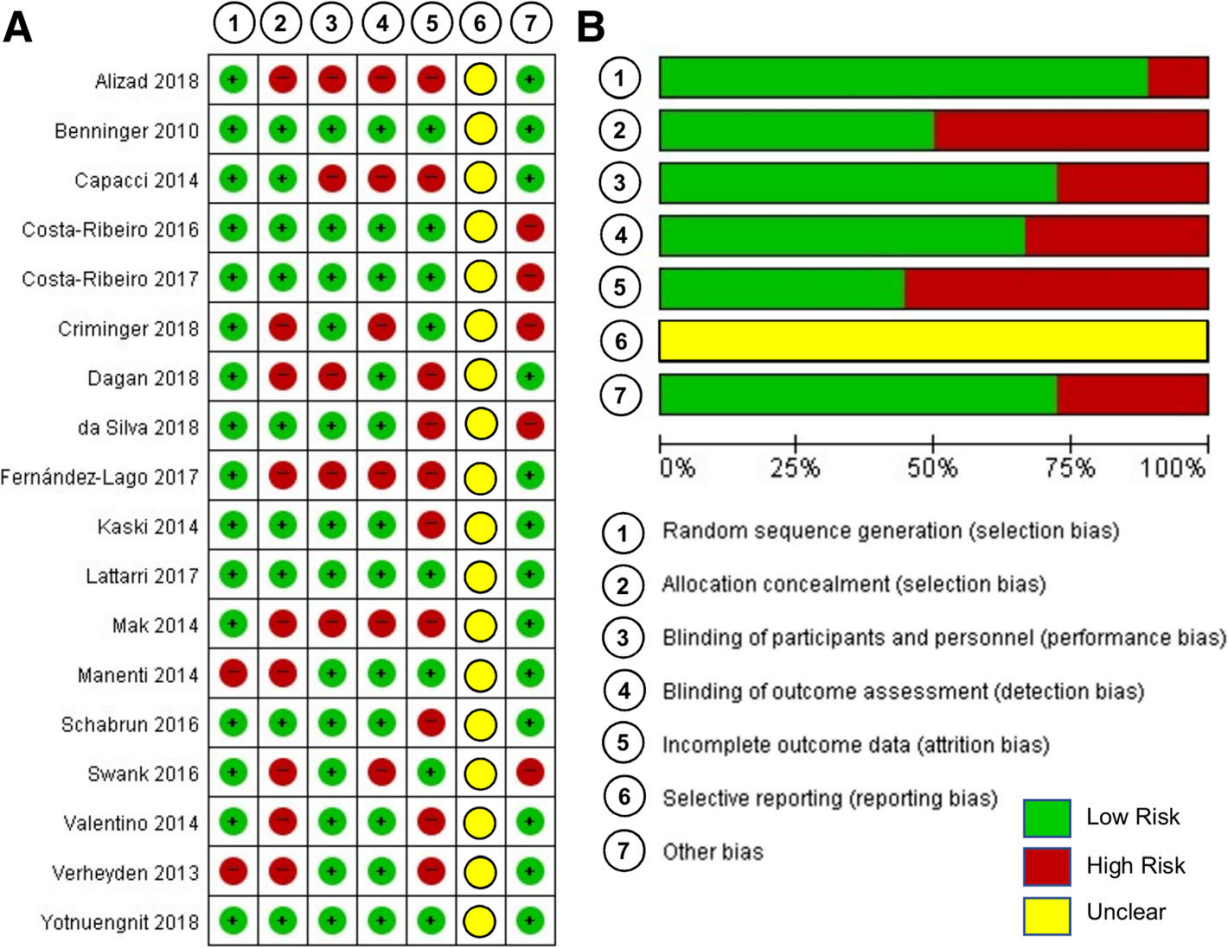
Cochrane risk of bias assessment. **a** Risk of bias summary and **b** Risk of bias graph

An average value of PEDro score was 7.7 (*SD* = 1.8), and this level indicates relatively good methodological quality across the included studies (Table 3). Moreover, we performed Cochrane’s methodological quality assessment for estimating the risk of bias within each study. Figure 2 displays the risk of bias summary and graph indicating relatively low risk of bias for each study except for the selective bias.

**Table 3:** PEDro score for methodological quality assessment

Items	Alizad [42]	Benninger [43]	Capacci [44]	Costa-Ribeiro [45]	Costa-Ribeiro [46]	Criminger [47]	da Silva [48]	Dagan [49]	Fernandez-Lago [50]
1. Specific eligibility criteria	0	1	0	1	1	1	1	1	1
2. Subjects random allocation	1	1	1	1	1	1	1	1	1
3. Allocation concealment	0	1	1	1	1	0	1	0	0
4. Similar groups at baseline	0	1	0	0	0	0	0	0	0
5. Blinding of all subjects	0	1	0	1	1	1	1	1	0
6. Blinding of all therapists	0	0	0	0	1	0	0	0	0
7. Blinding of all assessors (at least one key outcome)	0	1	0	1	1	0	1	1	0
8. Data measurement from more than 85% of the subjects initially allocated to groups (at least one key outcome)	1	1	1	1	1	1	1	1	1
9. All subjects received the treatment or control condition as allocated (at least one key outcome)	1	1	1	1	1	1	1	1	1
10. Between-group comparisons (at least one key outcome)	0	1	0	1	0	1	1	1	1
11. Point measures and measures of variability (at least one key outcome)	1	1	1	1	1	1	1	1	1
Total	4	10	5	9	9	7	9	8	6
Items	Kaski [51]	Lattari [52]	Mak [53]	Manenti [54]	Schabrun [55]	Swank [56]	Valentino [57]	Verheyden [58]	Yotnuengnit [59]
1. Specific eligibility criteria	1	1	0	1	1	1	1	1	1
2. Subjects random allocation	1	1	1	0	1	1	1	0	1
3. Allocation concealment	1	1	0	0	1	0	0	0	1
4. Similar groups at baseline	0	1	1	1	1	0	0	0	1
5. Blinding of all subjects	1	1	0	1	1	1	1	1	1
6. Blinding of all therapists	0	0	0	0	0	0	0	0	0
7. Blinding of all assessors (at least one key outcome)	1	1	0	1	1	0	1	1	1
8. Data measurement from more than 85% of the subjects initially allocated to groups (at least one key outcome)	1	1	1	1	1	1	1	1	1
9. All subjects received the treatment or control condition as allocated (at least one key outcome)	1	1	1	1	1	1	1	1	1
10. Between-group comparisons (at least one key outcome)	1	1	1	1	0	0	1	1	0
11. Point measures and measures of variability (at least one key outcome)	1	1	0	1	1	1	1	1	1
Total	9	10	5	8	9	6	8	7	9

### Meta-analytic results

**Fig. 3:**
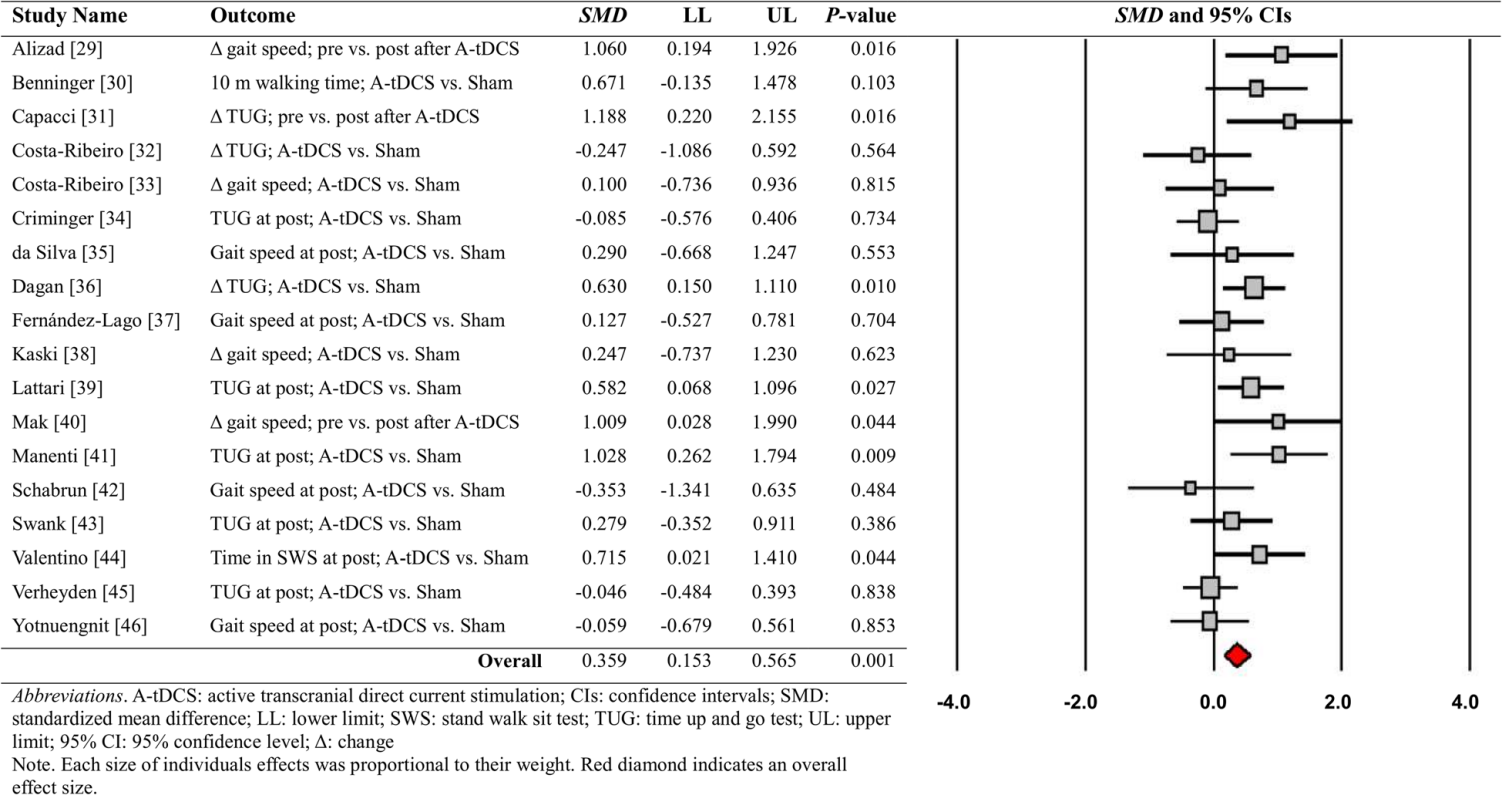
Meta-analytic findings for short-term treatment effects of tDCS

A random-effects model meta-analysis on the 18 comparisons from the qualified studies regarding the short-term treatment effects of tDCS revealed a significant overall effect size (*SMD* = 0.359; *SE* = 0.105; Variance = 0.011; 95% CI = 0.153–0.565; Z = 3.411; *P* = 0.001). The current level of overall effect size indicates a relatively small positive effect [60]. The individual weighted effect sizes across 18 comparisons are shown in Fig. 3. These findings indicate that applying tDCS slightly improved functional locomotion in people with PD, and these results were short-term treatment effects that appeared at the immediate posttest.

**Fig. 4:**
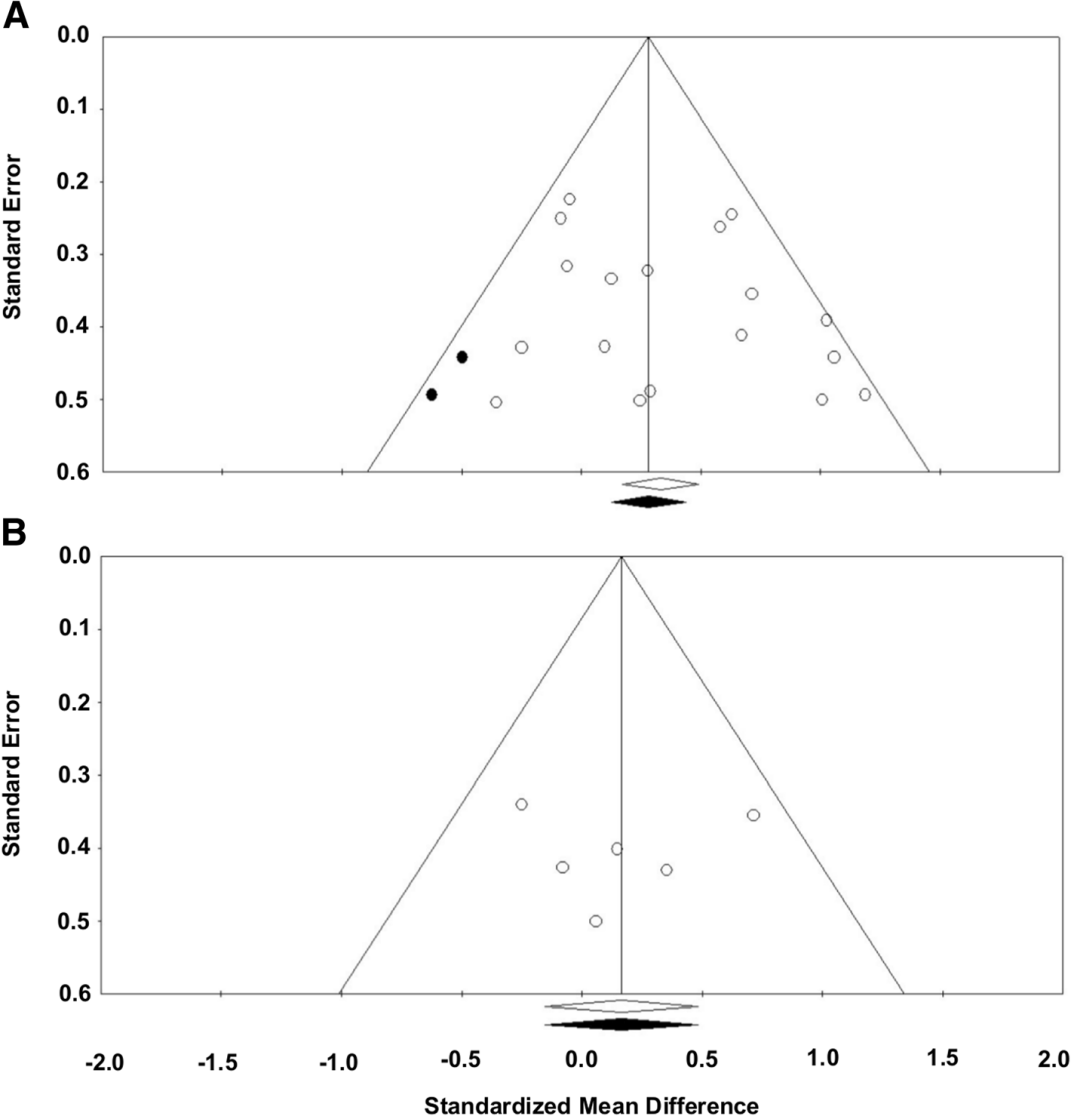
Publication bias assessments. **a** Short-term treatment effects of tDCS and **b** Long-term treatment effects of tDCS

For the short-term treatment effects of tDCS, heterogeneity test results were: (a) *Q*-statistics = 26.524 and *P*-value = 0.065, (b) *T*^2^ = 0.067, and (c) *I*^2^ = 35.907%. These findings indicate a relatively low level of individual effect size variability across the 18 comparisons. Moreover, publication bias was minimal because both the original and revised funnel plots after the trim and fill technique shows relatively similar overall effect sizes with two imputed values (Fig. 4a). This finding was additionally confirmed by two quantitative publication bias tests: (a) Egger’s regression test: intercept β_0_ = 1.281 and *P*-value = 0.250 and (b) Begg and Mazumdar rank correlation test: rank correlation τ = 0.163 and *P*-value = 0.343. Taken together, these meta-analytic findings indicate that the positive short-term effects of tDCS on functional locomotion had minimal heterogeneity and risk of bias across the included PD studies.

Moreover, we performed two additional sensitivity analyses to determine whether overall effect sizes were different across three functional locomotion tasks (gait speed vs. TUG time vs. stand walk sit test) and two functional locomotion quantification approaches (difference between active and sham tDCS groups at posttest vs. changes between baseline and posttest after active tDCS as compared with sham stimulation). The first sensitivity analysis revealed two significant positive effects from multiple comparisons: (a) nine gait speed comparisons: *SMD* = 0.307; *SE* = 0.151; Variance = 0.023; 95% CI = 0.011–0.603; Z = 2.030; *P* = 0.042, (b) eight TUG time comparisons: *SMD* = 0.365; *SE* = 0.164; Variance = 0.027; 95% CI = 0.044–0.686; Z = 2.229; *P* = 0.026, and (c) one stand walk sit test comparison: *SMD* = 0.715; *SE* = 0.354; Variance = 0.126; 95% CI = 0.021–1.410; Z = 2.019; *P* = 0.044. The second sensitivity analysis showed two significant positive effects: (a) 11 difference at posttest comparisons: *SMD* = 0.260; *SE* = 0.119; Variance = 0.014; 95% CI = 0.027–0.494; Z = 2.188; *P* = 0.029 and (b) seven changes from baseline to posttest comparisons: *SMD* = 0.555; *SE* = 0.191; Variance = 0.037; 95% CI = 0.180–0.929; Z = 2.902; *P* = 0.004.

**Fig. 5:**
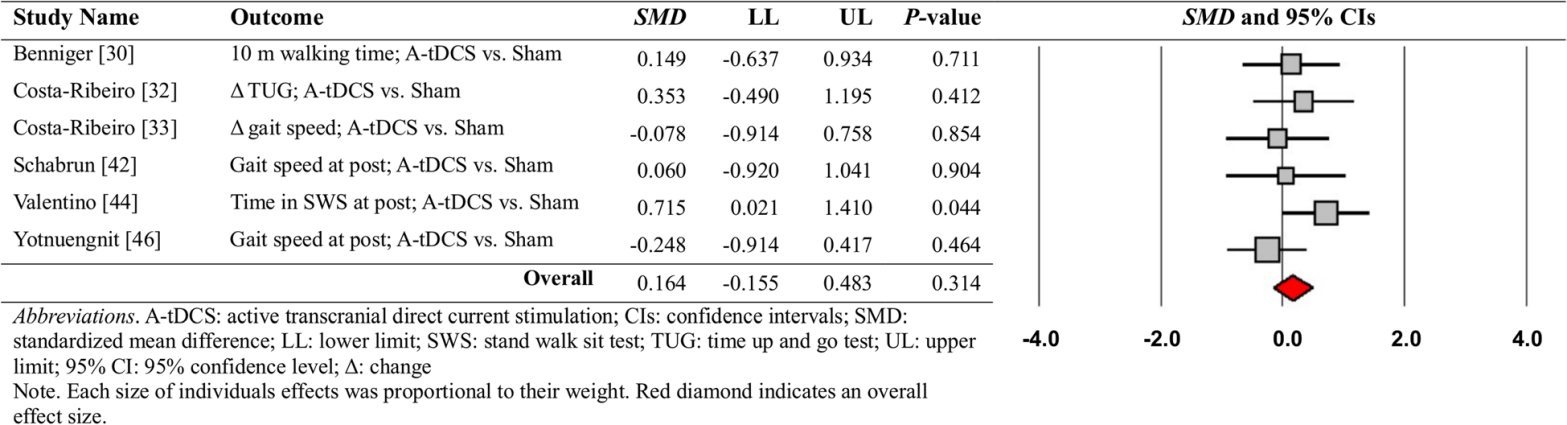
Meta-analytic findings for long-term treatment effects of tDCS

For long-term treatment effects of tDCS, a random effects model meta-analysis on six comparisons failed to show a significant overall effect size (*SMD* = 0.164; *SE* = 0.163; Variance = 0.026; 95% CI = − 0.155-0.483; Z = 1.007; *P* = 0.314; Fig. 5). Heterogeneity level for these findings was relatively small: (a) *Q*-statistics = 4.456 and *P*-value = 0.486, (b) *T*^2^ = 0.000, and (c) *I*^2^ = 0.00%. In addition, publication bias tests indicated minimal level of risk of bias across included studies: (a) identical overall effect sizes between original and revised funnel plots without any imputed value (Fig. 4b), (b) Egger’s regression test: intercept β_0_ = − 0.317 and *P*-value = 0.930 and (c) Begg and Mazumdar rank correlation test: rank correlation τ = 0.067 and *P*-value = 0.850. These findings revealed that tDCS protocols for people with PD did not reveal positive long-term effects on functional locomotion even with minimal variability and risk of bias across six studies.

Further, we conducted two similar sensitivity analyses for long-term effects of tDCS. First sensitivity analysis revealed no significant positive effects from multiple comparisons: (a) four gait speed comparisons: *SMD* = − 0.058; *SE* = 0.202; Variance = 0.041; 95% CI = − 0.455-0.339; Z = − 0.287; *P* = 0.774, (b) one TUG time comparison: *SMD* = 0.353; *SE* = 0.430; Variance = 0.185; 95% CI = − 0.490-1.195; Z = 0.821; *P* = 0.412, and (c) one stand walk sit test comparison: *SMD* = 0.715; *SE* = 0.354; Variance = 0.126; 95% CI = 0.021–1.410; Z = 2.019; *P* = 0.044. Second sensitivity analysis showed no significant positive effects: (a) four difference at posttest comparisons: *SMD* = 0.175; *SE* = 0.223; Variance = 0.050; 95% CI = − 0.262-0.613; Z = 0.786; *P* = 0.432 and (b) two changes from baseline to posttest comparisons: *SMD* = 0.136; *SE* = 0.303; Variance = 0.092; 95% CI = − 0.458-0.729; Z = 0.448; *P* = 0.654.

### Moderator variable analysis

A moderator variable analysis on comparisons for tDCS targeting multiple brain regions versus tDCS targeting a single brain region showed two significant positive overall effect sizes. We performed this moderator variable analysis for 18 short-term effect comparisons. Specific subgroup analyses found: (a) six multiple targeted areas: *SMD* = 0.527; *SE* = 0.194; Variance = 0.038; 95% CI = 0.146–0.908; Z = 2.711; *P* = 0.007 (heterogeneity tests: *Q*-statistics = 9.815 and *P*-value = 0.081; *T*^2^ = 0.11; *I*^2^ = 49.06%) and (b) 12 single targeted area: *SMD* = 0.272; *SE* = 0.126; Variance = 0.016; 95% CI = 0.026–0.518; Z = 2.165; *P* = 0.030 (heterogeneity tests: *Q*-statistics = 15.324 and *P*-value = 0.168; *T*^2^ = 0.05; *I*^2^ = 28.22%). These findings indicate that although both tDCS protocols revealed significant effect sizes, protocols stimulating multiple brain regions showed relatively more robust treatment effects on functional locomotion than single target tDCS protocols.

## Discussion

The current systematic review and meta-analysis investigated the treatment effects of tDCS interventions on functional locomotion in people with PD. Eighteen total comparisons from the qualified studies showed relatively small positive short-term effects (i.e., immediate posttest ≤24 h after final tDCS interventions) of tDCS, whereas six comparisons revealed no significant long-term effects (i.e., retention periods ≥4 weeks after final tDCS intervention) on functional locomotion in people with PD. Additionally, the moderator variable analysis found that applying tDCS on multiple targeted brain regions (e.g., M1 and PMC; M1 and prefrontal cortex; bilateral M1; bilateral DLPFC) may effectively improve functional locomotion of PD in comparison to tDCS protocols targeting a single brain region.

Our meta-analytic findings from 18 qualified studies revealed a significant immediate treatment effect on functional locomotion estimated by temporal gait measurements for 325 people with PD. Although the level of effect size was relatively small [60], these meta-analytic findings with more included studies extended prior findings that tDCS protocols may improve locomotion abilities in people with PD [29–31]. Some tDCS researchers proposed that facilitating cortical excitability using active tDCS may contribute to improvements in motor related symptoms of PD [22, 30]. Fregni and colleagues posited that cortical stimulation using tDCS may facilitate the neural connectivity in the cortical and subcortical networks (e.g., the basal ganglia-thalamocortical motor circuits) presumably improving degenerated functions of the basal ganglia in people with PD [22, 43, 61]. Moreover, the qualified studies in this meta-analysis frequently targeted motor and prefrontal cortices because of crucial role of motor and prefrontal cortical activations in locomotor performance of PD. Specifically, increased M1 activation patterns after anodal tDCS protocols were associated with motor improvements of PD [22]. During normal walking, people with PD were presumably dependent on cognitive control via increasing DLPFC activations for compensating their impairments in locomotion automaticity. However, given that more challenging walking may require greater DLPFC activation involvement in people with PD, anodal tDCS targeting prefrontal cortices may contribute to successful performance during functional locomotion tasks [23–25]. Finally, similar to previous rTMS studies that reported the release of dopamine in the caudate and putamen for healthy individuals [26, 27] and people with PD [28], tDCS interventions triggered on motor and prefrontal cortical regions possibly result in the release of dopamine contributing to functional locomotion improvements.

Our meta-analysis revealed no significant long-term treatment effects from six studies. Previous studies reported that cortical stimulation using tDCS facilitated neural plasticity and long-lasting effects for healthy individuals and people with other neurological disease [62–64]. The current meta-analysis indicated that beneficial effects of tDCS on functional locomotion in people with PD may be primarily manifested in immediate posttests, not in long-term delayed retention tests. These findings were consistent with previous suggestion that anodal tDCS of M1 could positively affect performance adaptation until only 3 h post training [65]. To elaborate the long-term effects of tDCS protocols on functional locomotion in people with PD, more studies with optimal stimulation protocols for exploring motor learning evidence should be necessary.

Interestingly, the moderator variable analysis revealed that tDCS protocols targeting multiple brain regions may provide better treatment effects on functional locomotion. In our meta-analysis, six studies targeted multiple brain regions such as PFC and M1, PFC, PMC, and M1, and bilateral DLPFC. Given that these motor and prefrontal cortical regions are key brain areas involved in dopaminergic circuits [22, 43, 61], stimulating simultaneously these regions presumably activated more neural connectivity and facilitated the release of dopamine in the caudate nucleus contributing to gait improvements [22, 66]. Specifically, stimulating DLPFC in addition to other motor cortical regions may improve functional locomotion via increasing extra-striatal dopamine release [66] and attenuating functional decoupling between the basal ganglia network and the cognitive control network involving DLPFC [67]. However, establishing specific tDCS protocols for optimizing functional locomotion rehabilitation is still required because of the inconsistent targeted brain regions in the current meta-analytic findings. Comparing treatment effects of a single brain region versus multiple regions targeted by tDCS protocols on PD functional locomotion rehabilitation may be an interesting research focus in the future studies.

Selecting an appropriate hemisphere site for tDCS may be an important issue for optimizing therapeutic effects of tDCS in people with PD. Conventional perspectives suggested that unilateral anodal tDCS targeting M1 of the more affected hemisphere may increase potential treatment effects on motor functions in people with PD [31, 54]. However, recent studies suggested that bilateral tDCS protocols (i.e., anodal tDCS on the more affected hemispheres and cathodal tDCS on the less affected hemisphere; anodal tDCS on the bilateral hemispheres) are presumably effective for facilitating lower limb functional recovery [43, 68]. Given that various lower limb abilities such as locomotion and postural control were highly associated with controlling the bilateral motor pathway from the affected and unaffected hemispheres [69], bilateral tDCS protocols may improve functional locomotion in people with PD. In this meta-analysis, five studies used anodal tDCS targeting bilateral motor and prefrontal cortical regions and six studies applied anodal tDCS on the central leg areas of M1. Future studies could consider diverse tDCS protocols including dual (motor and prefrontal cortices) and bilateral (more and less affected hemispheres) applications at clinical examination for PD motor recovery.

Despite the positive short-term effects of tDCS on functional locomotion identified in this meta-analysis, these findings are cautiously interpreted because of the possibility of dopaminergic medication suppressing functional locomotion improvements. Fourteen out of 18 total qualified studies in this meta-analysis applied tDCS interventions when people with PD were on medication although four studies did not report medication conditions. Importantly, perhaps the antiparkinson drugs confounded the effects of tDCS because of a ceiling effect [30]. Benninger and colleagues [43] compared tDCS effects on people with PD between “on” and “off” medications, and found greater reduction in bradykinesia during the “off” medication condition. Moreover, a prior study suggested a potential relationship between the required tDCS intensity and the intake of dopaminergic medication: 1 mA intensity of tDCS with “on” medication improved performance in people with PD whereas the same tDCS intensity revealed negative effects on gait performance with “off” medication [31]. Taken together, future studies should compare the effects of tDCS between medication conditions to tease apart the interaction effects of dopaminergic medication and tDCS on functional locomotion.

In addition, six out of 18 qualified studies reported freezing of gait (FOG) test results so that participants with severe FOG were excluded from the experiments. However, given that the remaining 12 studies in this meta-analysis did not report FOG conditions of people with PD, the heterogeneous inclusion and exclusion criteria for participants across the included studies may influence functional locomotion outcomes after tDCS protocols. Finally, our sensitivity analyses on the short-term effects of tDCS revealed comparable effect sizes across three functional locomotion tasks (gait speed vs. TUG time vs. stand walk sit test) and two functional locomotion quantification approaches (difference between active and sham tDCS groups at posttest vs. changes between baseline and posttest after active tDCS as compared with sham stimulation). However, these methodological heterogeneity issues may influence overall treatment effects of tDCS on functional locomotion in people with PD.

## Conclusions

In summary, the current systematic review and meta-analysis provided the evidence that tDCS interventions reveal short-term intervention benefits for functional locomotion in people with PD. However, the level of effect size was relatively small. Moreover, the treatment effects of active tDCS on functional locomotion of PD may increase when tDCS targeted multiple regions of motor and prefrontal cortices. These findings provide important clinical implications to researchers and clinicians in the utility of tDCS as a potential treatment protocol. To increase our understanding of tDCS treatment effects on functional locomotion, future studies should investigate optimal protocols including ideal targeted brain regions as well as medication conditions for functional locomotion rehabilitation in people with PD.

## Additional file

Additional file 1: PRISMA 2009 Checklist. (DOC 63 kb)

## Data Availability

The datasets generated during the current study are available from the corresponding author on reasonable request.

## Abbreviations

CI: Confidence interval
DBS: Deep brain stimulation
DLPFC: Dorsal lateral prefrontal cortex
M1: Primary motor cortex
NIBS: Non-invasive brain stimulation
PD: Parkinson’s disease
PFC: Prefrontal cortex
PMC: Premotor cortex
PRISMA: Preferred Reporting Items for Systematic Reviews and Meta-Analyses
SMA: Supplementary motor area
SMD: Standardized mean difference
tDCS: Transcranial direct current stimulation
