# Correction to: Does transcranial direct current stimulation improve functional locomotion in people with Parkinson’s disease? A systematic review and meta-analysis

**DOI:** 10.1186/s12984-019-0582-0

**Published:** 2019-11-14

**Authors:** Hyo Keun Lee, Se Ji Ahn, Yang Mi Shin, Nyeonju Kang, James H. Cauraugh

**Affiliations:** 1 Division of Sport Science, Neuromechanical Rehabilitation Research Laboratory, Incheon National University, Incheon, South Korea; 2 Vector Biomechanics Inc., Yongin, South Korea; 3 Sport Science Institute, Incheon National University, Incheon, South Korea; 4 Department of Applied Physiology and Kinesiology, University of Florida, Gainesville, USA

**Correction to: J NeuroEng Rehabil**

**https://doi.org/10.1186/s12984-019-0562-4**

In the original article [1], we mentioned that some study characteristics of the article by Dagan and colleagues [2] were unavailable. However, we realized that the authors provided the relevant information in their supplementary file. As such, we added participant characteristics (i.e., age = 68.8 ± 6.8, gender = 17 M, 3 F, PD duration = 9.0 ± 5.7, and UPDRS Part III at baseline = Total 39.7 ± 14.6) to Table 1, stimulation parameters (i.e., intensity = 3 mA, duration = 20 min, areas = 3 cm^2^) to Table 2, and methodological quality assessments (i.e., allocation concealment = 1 and Total score = 9) to Table 3. Based on the new information, we updated Fig. 2 with the corrected selection bias and performance bias results. Finally, we confirmed that these corrections did not change the meta-analytic findings in the original article.

**Table 1:** Participant characteristics

Study	Total N	Age (yrs)	Gender	PD Duration (yrs)	UPDRS Part III at Baseline	Medication	DBS Treatment	FOG Test
Alizad [42]	8	NA	Total: 3F, 5 M	NA	NA	NA	NO	NA
Benninger [43]	25	Total: 63.9 ± 8.7	Active: 4F, 9 M Sham: 5F, 7 M	Active: 10.6 ± 7.1 Sham: 9.1 ± 3.3	Active: 22.2 ± 8.7 Sham: 17.5 ± 8.0	On	NO	Patients with severe freezing or unable to walk 10 m were excluded
Capacci [44]	7	Total: 60.9 ± 9	Total: 4F, 3 M	Total: 16.8 ± 4.0	NA	NA	NO	NA
Costa-Ribeiro [45]	22	Active: 61.1 ± 9.1; Sham: 62.0 ± 16.7	Active: 3F, 8 M Sham: 4F, 7 M	Active: 6.1 ± 3.8 Sham: 6.3 ± 3.7	Active: 19.0 Sham: 19.1	On	NO	FOG-Q(> 15 points) were excluded
Costa-Ribeiro [46]	22	Active: 61.1 ± 9.1 Sham: 62.0 ± 16.7	Active: 3F, 8 M Sham: 4F, 7 M	Active: 6.1 ± 3.8 Sham: 6.3 ± 3.7	Active: 19.0 ± 4.9 Sham: 17.6 ± 5.1	On	NO	Patients were excluded when they presented severe freezing according the FOG-Q
Criminger [47]	16	Total: 68.1 ± 9.8	Total: 4F, 12 M	Total: 8.7 ± 9.8	Total: 23.4 ± 9.7	On	NO	NA
da Silva [48]	17	Active: 66.0 ± 5.0 Sham: 66.0 ± 10.0	Active: 4F, 4 M Sham: 3F, 6 M	Active: 6.0 ± 6.0 Sham: 5.0 ± 1.0	NA	NA	NO	NA
Dagan [49]	20	Total: 68.8 ± 6.8	Total: 17 M, 3 F	Total: 9.0 ± 5.7	Total: 39.7 ± 14.6	On	NO	FOG-Q: 20.5 ± 4.9 FOG-provoking test scores: 14.2 ± 8.00
Fernández-Lago [50]	18	Total: 56.7 ± 11.6	Total: 7F, 11 M	Total: 6.2 ± 3.7	Total: 21.17 ± 11.3	On	NO	NA
Kaski [51]	16	NA	NA	NA	NA	On	NO	Patients with severe freezing were excluded
Lattari [52]	17	Total: 67.2 ± 10.0	Total: 4F, 13 M	Total: 7.1 ± 2.7	Total: 18.0 ± 99.0	On	NO	NA
Mak [53]	18	NA	NA	NA	NA	NA	NO	NA
Manenti [54]	10	Total: 67.1 ± 7.2	Total: 4F, 6 M	Total: 8.1 ± 3.5	Total: 13.3 ± 5.7	On	NO	NA
Schabrun [55]	16	Active: 72.0 ± 4.9 Sham: 63.0 ± 11.0	Active: 8 M Sham: 6F, 2 M	Active: 6.9 ± 4.4 Sham: 4.6 ± 3.9	Active: 47.7 ± 7.5 Sham: 37.7 ± 9.8	On	NO	NA
Swank [56]	10	Total: 68.7 ± 10.2	Total: 2F, 8 M	Total: 7.9 ± 7.1	Total: 37.0 ± 12.9	On	NO	NA
Valentino [57]	10	Total: 72.3 ± 3.6	Total: 5F, 5 M	Total: 11.0 ± 4.9	Total: 32.0 ± 10.3	On	NO	FOG-Q: 15.3 ± 2.7
Verheyden [58]	20	NA	NA	Total: 9.0 ± 4.0	Total: 16.0 ± 5.0	On	NO	NA
Yotnuengnit [59]	53	Active: 68.2 ± 9.8 Sham: 62.7 ± 8.8	Active: 6F, 11 M Sham: 6F, 12 M	Active: 9.4 ± 5.3 Sham: 6.6 ± 3.6	Active: 11.9 ± 4.7 Sham: 11.2 ± 4.0	On	NO	NA

*Note*: Data for age and PD duration are mean ± standard deviation

*Abbreviations*: *Active* active tDCS protocols, *DBS* deep brain stimulation, *F* female, *FOG* Freezing of gait, *FOG-Q* Freezing of gait questionnaire, *M* male, *NA* not applicable, *PD Duration* time since PD diagnosis, *UPDRS* the Unified Parkinson’s Disease Rating Scale

**Table 2:** tDCS protocols

Study	Treatment	Session #	Active tDCS	Stimulation Site	Stimulation Parameters (Intensity, Duration, Areas)	Follow-Up Test
Alizad [42]	tDCS	3	A	M: Bi PMC & M1	1 mA, 20 min, 40 cm^2^	No
Benninger [43]	tDCS	8	A	M: Bi PFC, PMC, & M1 (separately)	2 mA, 20 min, 24.5 cm^2^	Yes (12wks)
Capacci [44]	tDCS	1	A	M: Bi PFC (separately)	2 mA, 20 min, NA	No
Costa-Ribeiro [45]	tDCS&GT	10	A	S: Central leg areas M1 (2 cm anterior to the vertex)	2 mA, 13 min, NA	Yes (4wks)
Costa-Ribeiro [46]	tDCS&GT	10	A	S: Central leg areas M1 (2 cm anterior to the vertex)	2 mA, 13 min, 35 cm^2^	Yes (4wks)
Criminger [47]	tDCS	3	A&C	M: Bi DLPFC (A-tDCS on LH & C-tDCS on RH)	2 mA, 20 min, 15 cm^2^	No
da Silva [48]	tDCS	1	A	S: Central leg areas M1 & SMA	2 mA, 15 min, 35 cm^2^	No
Dagan [49]	tDCS	2	A	M: M1 & LH-DLPFC	3 mA, 20 min, 3 cm^2^	No
Fernández-Lago [50]	tDCS&TT	1	A	S: leg area M1 of AH	2 mA, 20 min, 3.5 cm^2^	No
Kaski [51]	tDCS&PT	1	A	S: Central leg areas M1 (10–20% anterior to the vertex)	2 mA, 15 min, 40 cm^2^	No
Lattari [52]	tDCS	1	A	S: LH DLPFC	2 mA, 20 min, 35 cm^2^	No
Mak [53]	tDCS	5	A	S: M1	NA, 20 min, NA	No
Manenti [54]	tDCS	2	A	S: RH DLPFC	2 mA, 7 min, 35 cm^2^	No
Schabrun [55]	tDCS&GT	9	A	S: LH M1	2 mA, 20 min, 35 cm^2^	Yes (12wks)
Swank [56]	tDCS	1	A&C	M: Bi DLPFC (A-tDCS on LH & C-tDCS on RH)	2 mA, 20 min, NA	No
Valentino [57]	tDCS	5	A	S: Central leg areas M1	2 mA, 20 min, NA	Yes (4wks)
Verheyden [58]	tDCS	1	A	S: LH M1	1 mA, 15 min, NA	No
Yotnuengnit [59]	tDCS&PT	6	A	S: Central leg areas M1	2 mA, 30 min, 35 cm^2^	Yes (8wks)

*Abbreviations*: *A* anodal tDCS, *AH* affected hemisphere, *Bi* bilateral, *C* cathodal tDCS, *DLPFC* dorsolateral prefrontal cortex, *GT* gait training, *LH* left hemisphere, *M* multiple targeted brain regions, *M1* primary motor cortex, *NA* not applicable, *PFC* prefrontal cortex, *PMC* premotor cortex, *PT* physical training, *RH* right hemisphere, *S* single targeted brain region, *TT* treadmill training, *wks* weeks (retention period)

**Table. 3:** PEDro score for methodological quality assessment

Items	Alizad [42]	Benninger [43]	Capacci [44]	Costa-Ribeiro [45]	Costa-Ribeiro [46]	Criminger [47]	da Silva [48]	Dagan [49]	Fernandez-Lago [50]	Kaski [51]	Lattari [52]	Mak [53]	Manenti [54]	Schabrun [55]	Swank [56]	Valentino [57]	Verheyden [58]	Yotnuengnit [59]
1. Specific eligibility criteria	0	1	0	1	1	1	1	1	1	1	1	0	1	1	1	1	1	1
2. Subjects random allocation	1	1	1	1	1	1	1	1	1	1	1	1	0	1	1	1	0	1
3. Allocation concealment	0	1	1	1	1	0	1	1	0	1	1	0	0	1	0	0	0	1
4. Similar groups at baseline	0	1	0	0	0	0	0	0	0	0	1	1	1	1	0	0	0	1
5. Blinding of all subjects	0	1	0	1	1	1	1	1	0	1	1	0	1	1	1	1	1	1
6. Blinding of all therapists	0	0	0	0	1	0	0	0	0	0	0	0	0	0	0	0	0	0
7. Blinding of all assessors (at least one key outcome)	0	1	0	1	1	0	1	1	0	1	1	0	1	1	0	1	1	1
8. Data measurement from more than 85% of the subjects initially allocated to groups (at least one key outcome)	1	1	1	1	1	1	1	1	1	1	1	1	1	1	1	1	1	1
9. All subjects received the treatment or control condition as allocated (at least one key outcome)	1	1	1	1	1	1	1	1	1	1	1	1	1	1	1	1	1	1
10. Between-group comparisons (at least one key outcome)	0	1	0	1	0	1	1	1	1	1	1	1	1	0	0	1	1	0
11. Point measures and measures of variability (at least one key outcome)	1	1	1	1	1	1	1	1	1	1	1	0	1	1	1	1	1	1
Total	4	10	5	9	9	7	9	9	6	9	10	5	8	9	6	8	7	9

**Fig. 2:**
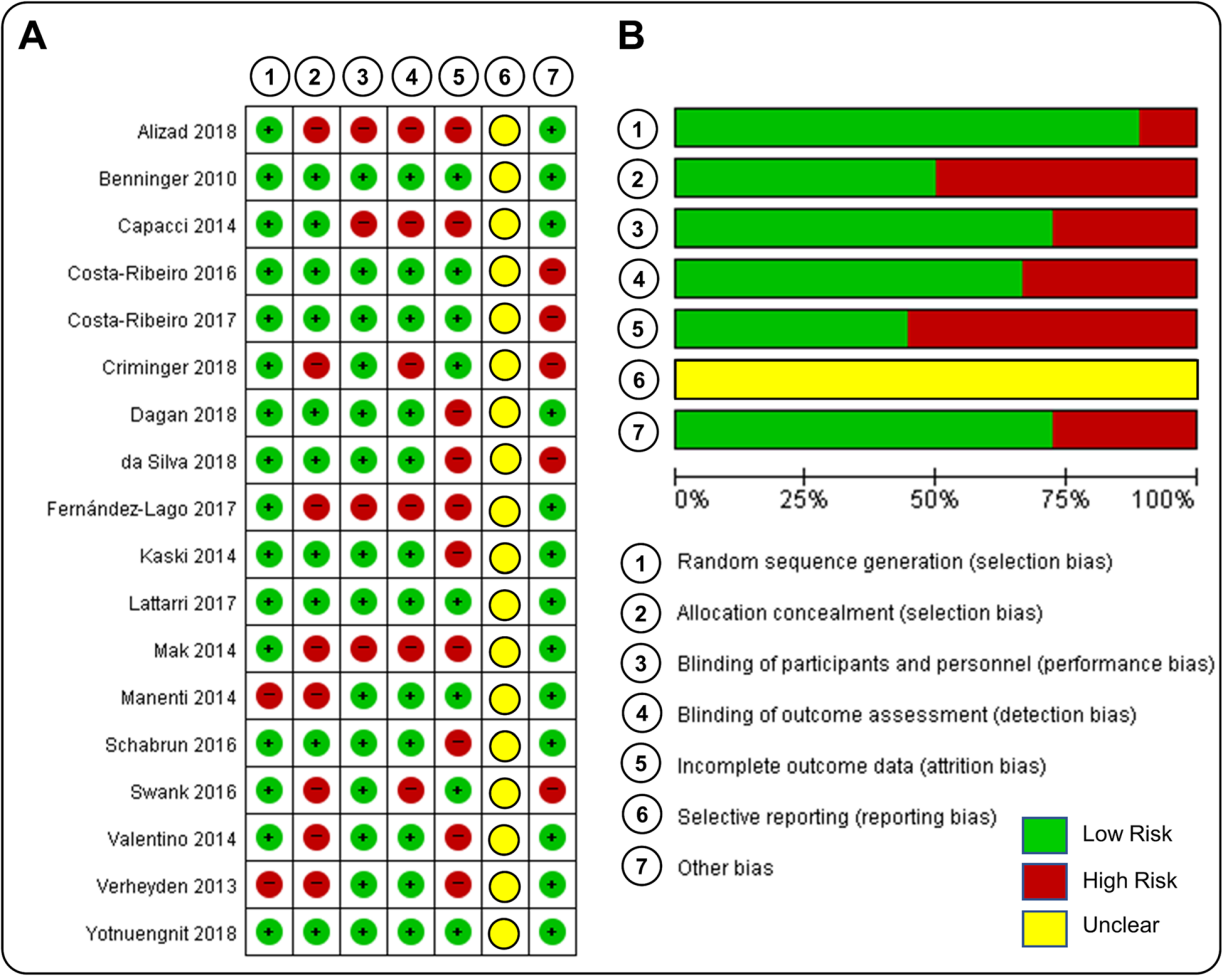
Cochrane risk of bias assessment. **a** Risk of bias summary and **b** Risk of bias graph
